# Proper Management of the Clinical Exposure Index Based on Body Thickness Using Dose Optimization Tools in Digital Chest Radiography: A Phantom Study

**DOI:** 10.3390/ijerph18105203

**Published:** 2021-05-13

**Authors:** Yongsu Yoon, Hyemin Park, Jungmin Kim, Jungsu Kim, Younghoon Roh, Nobukazu Tanaka, Junji Morishita

**Affiliations:** 1 Department of Radiological Science, Dongseo University, 47 Jurye-ro, Sasang-gu, Busan 47011, Korea; ysyoon@office.dongseo.ac.kr; 2 Department of Health and Safety Convergence Sciences, Korea University, 145 Anam-ro, Seongbuk-gu, Seoul 02841, Korea; dkviolet87@gmail.com; 3 Department of Radiological Technology, Daegu Health College, 15 Youngsong-Ro, Buk-gu, Daegu 41453, Korea; jungsu.kim73@gmail.com; 4 Department of Health Sciences, Faculty of Health Sciences, Kyushu University, 774 Motooka, Nishi-ku, Fukuoka 819-0395, Japan; nobukazu.tanaka@gmail.com (N.T.); Junjim@med.kyushu-u.ac.jp (J.M.)

**Keywords:** ALARA, diagnostic radiology, digital radiography, dose optimization, dose record, exposure index

## Abstract

In radiography, the exposure index (EI), as per the International Electrotechnical Commission standard, depends on the incident beam quality and exposure dose to the digital radiography system. Today automatic exposure control (AEC) systems are commonly employed to obtain the optimal image quality. An AEC system can maintain a constant incident exposure dose on the image receptor regardless of the patient thickness. In this study, we investigated the relationship between body thickness, entrance surface dose (ESD), EI, and the exposure indicator (S value) with the aim of using EI as the dose optimization tool in digital chest radiography (posterior–anterior and lateral projection). The exposure condition from the Korean national survey for determining diagnostic reference levels and two digital radiography systems (photostimulable phosphor plate and indirect flat panel detector) were used. As a result, ESD increased as the phantom became thicker with constant exposure indicator, which indicates similar settings to an AEC system, but the EI indicated comparatively constant values without following the tendency of ESD. Therefore, body thickness should be considered under the AEC system for introducing EI as the dose optimization tool in digital chest radiography.

## 1. Introduction

Radiology has contributed to the development of modern medicine by enabling diagnosis without using invasive methods since the discovery of X-ray by Rontgen in 1895 [1]. The radiographic system has evolved from the analog screen-film system to computed radiography (CR), which is considered to be a bridge that links the analog system to a fully digital system and digital radiography (DR) system, such as indirect/direct flat panel detector (FPD). Earlier, it was important to adjust an optimal exposure condition for obtaining proper image quality, as the radiological technologist cannot check the quality of the image until the film is developed due to the characteristics of the film/screen, such as the sensitivity [1,2,3]. However, digital radiography systems with wide dynamic range have made it possible to take radiographs of intended quality to introduce the post-image processing and lookup table, which can offer proper sensitivity of an image [2,3,4].

Digital image processing can provide convenience to both the operator and patients, but there is a concern that a digital radiographic system can increase the exposure dose [2]. Previous studies have revealed that radiological technologists tend to take a radiograph with a higher exposure dose to avoid re-examination. Seo et al. reported that the entrance surface dose (ESD) of a digital radiography system was 55.25% higher than that of the analog system in general radiographic examinations in Korea [2,5]. Therefore, it is essential to manage and control the exposure dose of the digital radiography system for patient safety regarding radiation protection.

To manage the radiation exposure in the radiology department, there have been several studies on the introduction of an automatic radiation exposure dose monitoring system using a hospital information system and a radiology information system through digital information communication of medicine (DICOM) header of image [6,7].

Several methods were available to record the exposure dose for monitoring systems such as the kerma-area product meter and numerical dose determination (NDD) method [8,9,10]. However, these methods have a complicated process to measure and calculate the entrance exposure dose. Thus, it was difficult to utilize these methods in a clinical situation.

**Figure 1:**
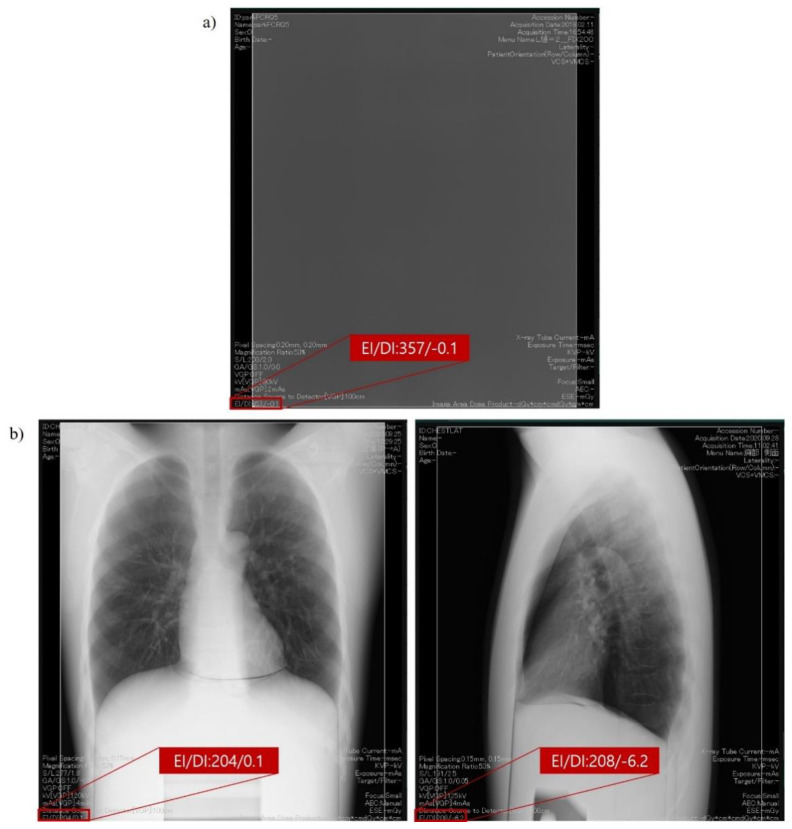
EI and DI* displayed on the digital system console: (**a**) EI as per IEC standards; (**b**) clinical EI of chest PA and LAT examination (*EI_T_ is not calibrated at this time, thus DI is arbitrary value in this figure).

The International Electrotechnical Commission (IEC) has introduced the concept of exposure index (EI) to manage the radiation exposure in the field of digital radiography using digital radiation detectors, such as a photostimulable phosphor (PSP) plate, and FPD [11]. EI has some merits to manage radiation exposure compared to a conventional exposure indicators such as the S value [12]. The S value, which represents the numeric value of the incident radiation exposure on the detector plane in a digital system, is similar to the sensitivity of an analog system [12]. According to the IEC recommendation, EI should have a linear relationship with the exposure dose and it should not be modified after the examination. EI, as per IEC standards, indicated that it could be adapted to quality assurance and the control of digital radiation detectors (Figure 1a). Moreover, the target exposure index (EI_T_) and deviation index (DI) were also introduced for the optimization and management of radiation exposure [11].

However, there is a gap between the measurement condition of EI, as per IEC standards, and that of clinical situations such as the presence of an object (patient) and varying beam quality. Therefore, manufacturers have introduced the clinical exposure index according to each general radiographic examination (hereinafter referred to as “clinical EI”, Figure 1b). Compared to traditional exposure indicators in digital radiography systems, the clinical EI is proportional to incident exposure dose and cannot be modified after the examination regardless of the manufacturer and type of digital radiography system [13].

Previous studies have reported the usefulness of EI, as per IEC standards, which can be achieved using a quality control tool and an effective radiation dose monitoring system. Park et al. investigated the possibility of using it as a quality control tool by using displayed EI, as per IEC standards, and also revealed that the usefulness of clinical EI, EI_T_, and DI, which could be recorded in DICOM header tags for monitoring the ESD based on the national diagnostic reference levels (DRLs) [13,14]. In clinical situations, there are several trials that have introduced the clinical EI, EI_T_, and DI to optimize medical radiation use [15,16,17].

However, the calculation of EI depends on the beam quality and the image receptor dose. There is a possibility that EI cannot reflect the ESD, as the size of the patient varies [14]. Nowadays, an automatic exposure control (AEC) system is commonly employed to adjust an optimal sensitivity of radiograph regardless of the patient size, thus, the tube current product time (mAs) would vary with the size of the patient to maintain the identical quality of radiograph [18]. Careful measurement and estimation of ESD are important for the establishment of DRLs and the evaluation of exposure risk to the patient [19,20]. It is essential to identify whether or not the clinical EI reflects the actual ESD for clinical usage as a dose optimization tool. Therefore, we investigated the relationship between the clinical EI and ESD with various phantom thickness in chest radiography to use the clinical EI as the exposure dose optimization tool through convergence of digital radiography images, medical information, and measured exposure dose.

## 2. Materials and Methods

### 2.1. Equipment

For this study, we employed a CR system and a DR system from a single manufacturer. The specifications of each system were as follows. CR system: PROFECT CS, photostimulable phosphor plate: ST-VI, single-side reading system; FUJIFILM Medical Co. Ltd., Tokyo, Japan; DR system specifications: CALNEO Smart C47, an indirect-FPD system, pixel size: 150 μm, scintillator material: CsI: Tl, FUJIFILM Medical Co., Ltd., Tokyo, Japan. Both systems were equipped with an automatic display function for the EI as per IEC framework standards and clinical EI. An X-ray generator (UD 150L-40, Shimadzu, Co. Ltd., Kyoto, Japan) and an X-ray tube assembly (P-380DE-85, Shimadzu, Co. Ltd.) were used. A semiconductor detector (RaySafe X2, Unfors RaySafe, Billdal, Sweden) was used to measure the air-kerma where the image receptor was placed.

### 2.2. Exposure Index as per the International Electrotechnical Commission Standard Framework for Quality Assurance and Control of Digital Radiography Systems Used in This Study

Before using the EI in a clinical situation, the user should identify the characteristics of the digital system, such as whether the uncertainty of the EI value in calibration conditions is according to IEC 62494-1 [11,14]. The IEC specifies that EI should be calculated by uniformly irradiating the relevant image region with the radiation quality (RQA) 5 in the absence of a subject and using the central 10% of the relevant image region as the value of interest (VOI) [11]. The EI value under the calibration condition is defined as the following Equation (1):

EI = C_0_ × K_cal_ (1)

where C_0_ is the correction factor as 100 μGy^−1^ and K_cal_ is incident air-kerma (μGy) measured under the calibration conditions [11]. In this study, this EI is referred to as the “reference EI”. The IEC also recommends that the EI under the calibration conditions provided by the manufacturer of the digital system should have an uncertainty of less than 20% compared to the reference EI [11].

The RQA5 was satisfied with 6.73 mmAl of the half-value layer (HVL) measured under conditions using the tube voltage of 74 kVp, a field size of 35.4 × 43.0 cm^2^, source to image receptor distance (SID) of 150 cm, and an additional filtration of 21 mm aluminum. The tube current was fixed at 200 mA and the exposure time was increased to 10 to 250 msec (2 to 50 mAs). Moreover, the incident air-kerma, which is the incident on the detector, was measured by attaching a lead to the back of the detector of the dosimeter to avoid the influence of backscattered radiation. We recorded the incident air-kerma and the EI value displayed on the console which is provided by the manufacturer. To evaluate the uncertainty of displayed EI values, the measured incident air-kerma was multiplied by C_0_ of 100 μGy^−1^, and it was compared with the displayed EI values. All of the digital system (CR and DR) used in this study were evaluated in the same condition.

### 2.3. Exposure Condition and Geometry for Obtaining Clinical Exposure Index with Various Thickness of Phantom

**Figure 2:**
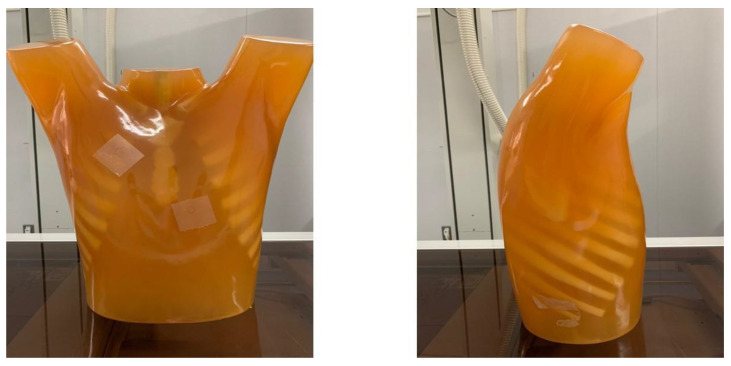
An anthropomorphic chest phantom used in this study.

To obtain the clinical EI of chest radiography, the exposure condition based on the latest Korean national survey to update the national DRLs for general radiographic examinations in 2019 was used [21]. The tube voltage was 120 kVp and SID was 180 cm for both chest posterior-anterior (PA) and lateral (LAT) examination (Table 1). These exposure conditions are the result of the statistical analysis of data collected nationwide through a questionnaire survey in clinical institutions that have a radiology department [21,22]. The anti-scatter grid with a grid ratio of 10:1 was used. We used an anthropomorphic chest phantom (Multipurpose Chest Phantom N1 “LUNGMAN”, Kyoto Kagaku, Co., Ltd., Kyoto, Japan). This phantom represents the upper torso part of the human body, including vertebrae, clavicles, ribs, and sternum. The Hounsfield numbers of the materials used in this phantom for soft tissue and organs, bones, and joints were equivalent to those of the corresponding real human organs (Figure 2) [23]. The dosimeter for measuring the ESD was placed directly on top of the phantom, such that it faced the direction of X-ray irradiation.

**Figure 3:**
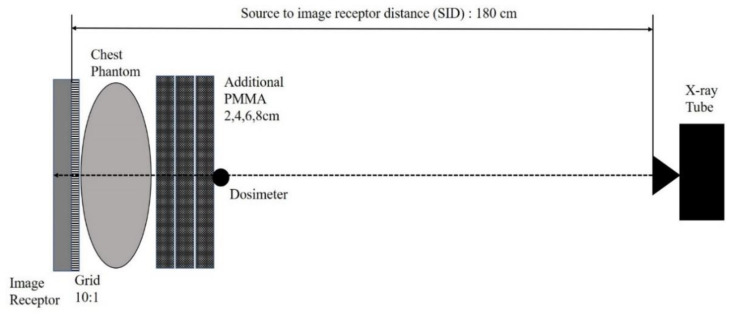
Scheme of experimental geometry in this study.

To identify the effect of body size on clinical EI, we set up the geometry shown in Figure 3. The chest phantom was placed in front of the image receptor and the polymethyl-methacrylate (PMMA) was added from 2 cm to 8 cm, which represents the variation of patient body thickness in a clinical situation. To ensure a similar condition of operation for the AEC system, we set the sensitivity indicator, namely, “S value” as 200 with ±10% of allowable fluctuation, for maintaining identical image quality and incident exposure dose to the image receptor [24]. As the thickness of the phantom varied, mAs also varied to ensure the same incident exposure dose to the image receptor.

## 3. Results

**Figure 4:**
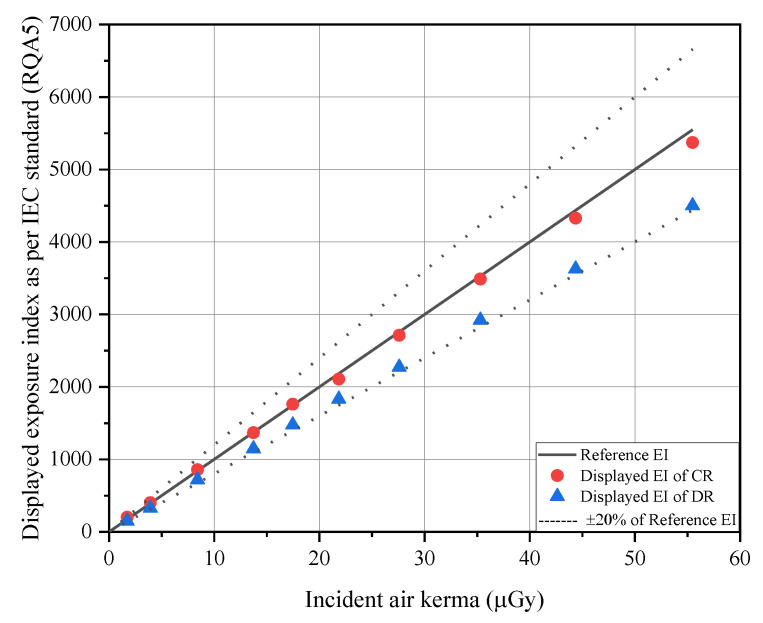
Relationship between reference EI and displayed EI.

Table 2 shows the reference EI determined through the incident air-kerma measured in RQA5, which is the calibration beam quality as per IEC and the displayed EI values by each system under the same exposure condition. Table 2 also shows the uncertainty of the EI displayed for each system relative to the reference EI, and their relationship is shown in Figure 4. In all incident air-kerma ranges, uncertainties of displayed EI were 0.34% to 17.59% for CR and 14.27% to 18.90% for DR.

**Figure 5:**
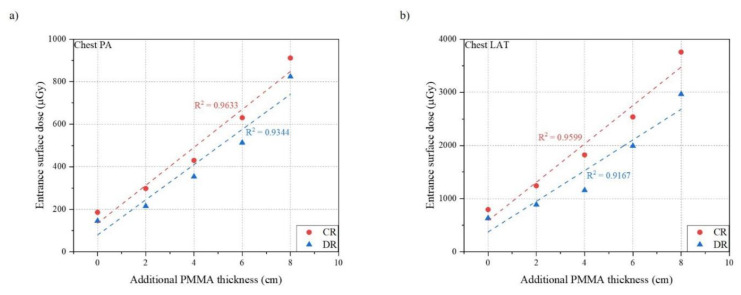
Relationship between phantom thickness and entrance surface dose: (**a**) Chest PA; (**b**) Chest LAT.

**Figure 6:**
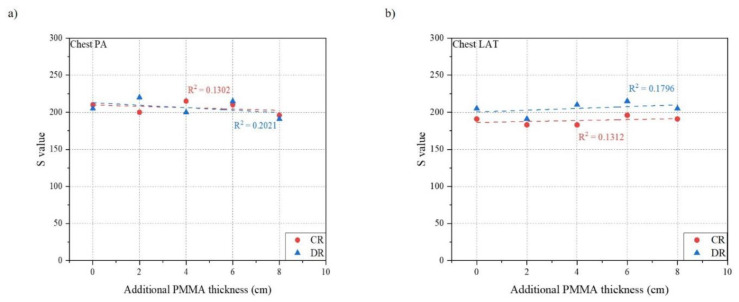
Relationship between phantom thickness and S value: (**a**) Chest PA; (**b**) Chest LAT.

**Figure 7:**
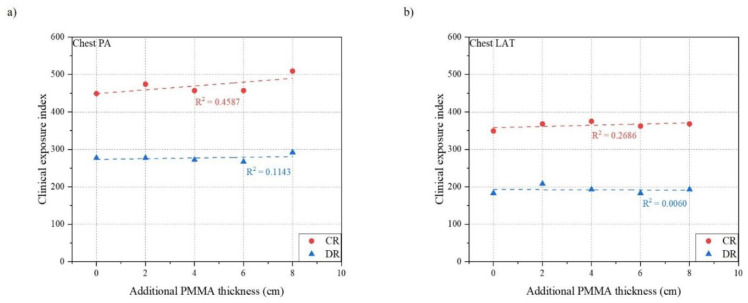
Relationship between phantom thickness and clinical exposure index: (**a**) Chest PA; (**b**) Chest LAT.

Table 3 and Table 4 show the result of measurements for chest PA and LAT examination using both CR and DR systems. Figure 5, Figure 6 and Figure 7 indicates the relationship between the phantom thickness and the ESD, S value, and EI.

For chest PA examination, the ESD was proportional to the thickness of the phantom which increased in the range of 186.1 to 911.0 μGy for CR and 145.8 to 823.5 μGy for DR. The Pearson correlation coefficients were 0.9633 for CR and 0.9344 for DR which shows the strong correlation between the ESD and the thickness of the phantom. On the contrary, both S value and EI for CR and DR showed a constant relationship, even if the phantom thickness increased. All Pearson correlation coefficients were under 0.5, which indicated a very weak correlation with the phantom thickness between the S value and EI.

In the case of chest LAT examination, the tendencies of relationships between the phantom thickness and the ESD, S value, and EI were similar to those of chest PA examination. The ESD increased as the phantom thickness increased in the range of 796.1 to 3757.0 μGy for CR and 635.4 to 2965.7 μGy for DR. The Pearson correlation coefficients were 0.9599 for CR and 0.9167 for DR, which showed a strong relationship between the ESD and the phantom thickness. However, both the S value and EI for CR and DR showed a constant relationship even if the phantom thickness increased. All Pearson correlation coefficients were under 0.3, which indicated a very weak correlation with the phantom thickness between the S value and EI.

## 4. Discussion

In this study, we investigated the effect of body thickness on clinical EI, to introduce the clinical EI as a dose optimization tool in chest radiography.

First, the EI provided by manufacturers, as per IEC standards, should have an uncertainty of less than 20% compared to the reference EI in RQA5, which is the calibration beam quality [11]. The X-ray generator used in this study indicated HVL of 6.73 mmAl when irradiated with a tube voltage of 74 kVp, and satisfied RQA5 as the corresponding condition. Subsequently, the reference EI, which is the value obtained by multiplying the incident air-kerma measured using the RQA5 conditions by C_0_ (100 μGy^−1^), was compared with the EI displayed on the console of the digital system. Therefore, the uncertainties of both systems were within 20% in all incident air-kerma ranges, and thus we have determined that the EIs of both systems can be used as dose optimization tools. Displayed EI, as per the IEC standards used in this study, is automatically calculated and displayed by modality. According to a previous study, the uncertainty of displayed EI was higher than that of manually calculated EI, but both EIs indicated allowable uncertainty (<20%), as per IEC standards. This discrepancy occurred due to the differences of digital characteristics between manually calculated and automatically displayed EI because of the differences of the digital characteristic curve used for the calculation [14]. However, the IEC standard recommended an EI uncertainty of under 20%, thus, if the displayed EI satisfied within the range of uncertainty (<20%), the digital radiation detector would be regarded as under good quality-controlled status [11,13,14]. Moreover, it is reported that prior to using the EI, understanding the characteristics of digital system is necessary [13,14]. We used two different digital systems from the same manufacture, but the clinical EI of the chest examinations did not indicate the same value. In this study, the clinical EI of the CR system showed a higher value than that of the DR system. However, this does not mean that higher clinical EI indicates higher incident X-rays on the detector plane even though it was calculated by the same processing console. According to previous studies, several reasons such as the type of scintillation materials used in the detector, calibration status conducted by the manufacturer, and the calculation method of clinical EI for various examinations affect clinical EI [14,25]. Therefore, operators in the radiology department should understand that even though the same incident air-kerma was exposed in both digital systems under the same exposure conditions, the displayed EI, as per IEC standards, and the clinical EI value were found to be different. Therefore, the users who use the digital system should consider these characteristics.

The ESD is commonly employed for evaluating the patient exposure dose and the risk of radiation exposure in medicine [10,19]. To achieve the justification and optimization concept on the usage of medical radiation, the tendency of ESD with various phantom thicknesses should be reflected in the clinical EI. According to the results, ESD increased as the phantom thickness increased in both chest PA and LAT projection, under an AEC system which maintained a S value of approximately 200. Moreover, the correlation between ESD and the phantom thickness was very strong because the source to patient distance became shorter with greater exposure dose to maintain identical image quality regardless of the phantom thickness. However, the clinical EI of both chest PA and LAT projection did not indicate a similar tendency with ESD, and the phantom distance compared with those of ESD. Takaki et al. reported that the EI, as per the IEC standards framework, depends on the PMMA phantom thickness, and thus EI should be introduced cautiously for children and thin patients [26]. Sánchez et al. proposed that the thickness-based techniques for portable pediatric abdomen radiography should be used to reduce the variability in EI because default pediatric protocols do not adequately characterize the patient size, which is the principal determinant of proper imaging technique [27]. Our measurements using the clinical EI in digital systems indicated similar results as those of a previous study, that EI cannot reflect patient thickness. There have been many studies on introducing EI to manage patient exposure dose and image quality, but there is a possibility of underestimating patient dose if operators run the dose managing tool using EI without any consideration of patient thickness. Park et al. also reported that the clinical EI_T_ for chest and abdomen radiography should be updated periodically as the change of exposure condition [13]. However, the interventional reference point was defined by the IEC to represent the patient skin level for evaluating and monitoring the patient dose in multi-modality [28]. Moreover, the reference thickness of the patient has been already recommended to establish the DRLs in general radiographic examinations [19]. Previous studies have reported that DRLs of chest and abdomen radiography can be used for setting the clinical EI_T_ to manage exposure dose [13]. Therefore, the reference point for the clinical EI should be established based on the patient thickness, beam quality, and the type of imaging system. The operators who utilize the clinical EI, EI_T_, and DI as dose monitoring tools should understand the discrepancy between ESD and the clinical EI in a clinical situation.

## 5. Conclusions

The clinical EI in chest radiography cannot reflect the tendency of ESD, as the phantom thickness increases with an AEC system. Therefore, operators should be cautious when evaluating the exposure dose using the EI, EI_T_, and DI when the EI is stable, regardless of patient thickness in clinical situations.

## Figures and Tables

**Table 1:** The exposure condition based on the latest Korean national survey to update the national DRLs for general radiographic examinations in 2019.

Examination	Tube Voltage (kVp)	Tube Current Time Product (mAs)	SID (cm)	Field Size (cm × cm)
Chest PA	120	4 (2.8–7.4)	180	42 × 43
Chest LAT	120	10.6 (8–16)	180	42 × 43

**Table 2:** Displayed EI and uncertainty from reference EI in RQA5.

mAs	Incident Air-Kerma (μGy)	Reference EI ^(1)^	Displayed EI (Uncertainty %)	
			CR	DR
2	1.73	173	203 (+17.59)	148 (−14.27)
4	3.93	393	403 (+2.65)	326 (−16.96)
8	8.42	842	857 (+1.84)	718 (−14.68)
12.6	13.74	1374	1369 (−0.34)	1147 (−16.50)
16	17.47	1747	1761 (+0.82)	1475 (−15.55)
20	21.84	2184	2108 (−3.49)	1831 (−16.18)
25	27.59	2759	2711 (−1.75)	2272 (−17.66)
32	35.30	3530	3488 (−1.20)	2922 (−17.23)
40	44.36	4436	4328 (−2.43)	3627 (−18.24)
50	55.49	5549	5371 (−3.20)	4500 (−18.90)

^(1)^ Defined as the product of the incident air-kerma and C_0_ (100 μGy^−1^).

**Table 3:** Result of tube current time product, S value, entrance surface dose, and clinical exposure index for chest radiography (PA projection).

Body Thickness	mAs		S Value		ESD (μGy)		Clinical EI	
	CR	DR	CR	DR	CR	DR	CR	DR
Only Phantom	3.6	2.8	210	205	186.1	145.8	449	277
+2 cm PMMA	5.6	4	200	220	298.2	215.2	474	277
+4 cm PMMA	8	6.4	215	200	430.2	353.8	457	272
+6 cm PMMA	11.2	9	210	215	630.5	513.0	457	267
+8 cm PMMA	16	14.4	196	191	911.0	823.5	509	292

**Table 4:** Result of tube current time product, S value, entrance surface dose, and clinical exposure index for chest radiography (LAT projection).

Body Thickness	mAs		S Value		ESD (μGy)		Clinical EI	
	CR	DR	CR	DR	CR	DR	CR	DR
Only Phantom	12.8	10	191	205	796.1	635.4	349	183
+2 cm PMMA	17.9	12.6	183	191	1243.0	888.3	368	208
+4 cm PMMA	25.6	16	183	210	1822.7	1158.7	375	193
+6 cm PMMA	35.2	27.5	196	215	2537.3	1991.7	362	183
+8 cm PMMA	51.2	40	191	205	3757.0	2965.7	368	193

## Data Availability

Data can be requested from the corresponding author.
